# Integrating genomic-enabled prediction and high-throughput phenotyping in breeding for climate-resilient bread wheat

**DOI:** 10.1007/s00122-018-3206-3

**Published:** 2018-10-19

**Authors:** Philomin Juliana, Osval A. Montesinos-López, José Crossa, Suchismita Mondal, Lorena González Pérez, Jesse Poland, Julio Huerta-Espino, Leonardo Crespo-Herrera, Velu Govindan, Susanne Dreisigacker, Sandesh Shrestha, Paulino Pérez-Rodríguez, Francisco Pinto Espinosa, Ravi P. Singh

**Affiliations:** 1 International Maize and Wheat Improvement Center (CIMMYT), Postal 6-641, 06600 Mexico, D.F. Mexico; 2 Facultad de Telemática, Universidad de Colima, Colima, 28040 Mexico; 3 Department of Plant Pathology and Agronomy, Wheat Genetics Resource Center, Kansas State University, Manhattan, KS 66506 USA; 4 Colegio de Postgraduados, Montecillo, Edo. de México 56230 Mexico; 5 Campo Experimental Valle de México INIFAP, Chapingo, Edo. de México 56230 Mexico

## Abstract

Genomic selection and high-throughput phenotyping (HTP) are promising tools to accelerate breeding gains for high-yielding and climate-resilient wheat varieties. Hence, our objective was to evaluate them for predicting grain yield (GY) in drought-stressed (DS) and late-sown heat-stressed (HS) environments of the International maize and wheat improvement center’s elite yield trial nurseries. We observed that the average genomic prediction accuracies using fivefold cross-validations were 0.50 and 0.51 in the DS and HS environments, respectively. However, when a different nursery/year was used to predict another nursery/year, the average genomic prediction accuracies in the DS and HS environments decreased to 0.18 and 0.23, respectively. While genomic predictions clearly outperformed pedigree-based predictions across nurseries, they were similar to pedigree-based predictions within nurseries due to small family sizes. In populations with some full-sibs in the training population, the genomic and pedigree-based prediction accuracies were on average 0.27 and 0.35 higher than the accuracies in populations with only one progeny per cross, indicating the importance of genetic relatedness between the training and validation populations for good predictions. We also evaluated the item-based collaborative filtering approach for multivariate prediction of GY using the green normalized difference vegetation index from HTP. This approach proved to be the best strategy for across-nursery predictions, with average accuracies of 0.56 and 0.62 in the DS and HS environments, respectively. We conclude that GY is a challenging trait for across-year predictions, but GS and HTP can be integrated in increasing the size of populations screened and evaluating unphenotyped large nurseries for stress–resilience within years.

**Electronic supplementary material:**

The online version of this article (10.1007/s00122-018-3206-3) contains supplementary material, which is available to authorized users.

## Introduction

In the face of global food security and climate change, breeding bread wheat (*Triticum aestivum* L.) with high-yield potential and improved resilience to stressed environments is crucial (Curtis and Halford 2014; Reynolds et al. 2016). Although conventional breeding has led to substantial improvements in wheat grain yield (GY), breeders are constantly challenged with extreme weather conditions and increasing drought and heat-stressed environments (Trnka et al. 2014; Tack et al. 2015; Mäkinen et al. 2017; Zampieri et al. 2017). Hence, breeding for tolerance to abiotic stress is one of the key goals of the International Maize and Wheat Improvement Center (CIMMYT) in several mega-environments (Braun et al. 1996). It was estimated that about 55% of the wheat-sown area in CIMMYT’s target countries is threatened by the periodical occurrence of drought (Trethowan et al. 2002). Similarly, continuous heat stress is a severe constraint in one of CIMMYT’s mega-environments including south and central India (Cossani and Reynolds 2012), and it is expected that 51% of the Indo-Gangetic plains might be reclassified as a heat-stressed mega-environment by 2050 (Ortiz et al. 2008). In addition, it was also predicted that global wheat production would decrease between 4.1 and 6.4%, for every °C rise in temperature (Liu et al. 2016).

Breeding bread wheat for resilience to drought and heat stress involves identifying the key physiological and genetic drivers of GY in these stressed environments. For drought stress, physiological traits that increase water use, water-use efficiency, and partitioning of carbon to the grain are known to be involved in adaptation (Olivares-Villegas et al. 2007; Reynolds and Tuberosa 2008; Lopes et al. 2014). Similarly, for heat stress, traits that increase light interception, radiation use efficiency, and total assimilate partitioning are the principal drivers of GY (Farooq et al. 2011; Cossani and Reynolds 2012). Considering the genetic basis of GY under drought and heat stress, several quantitative trait loci (QTL) have been identified in previous studies (Kirigwi et al. 2007; Pinto et al. 2010; Bennett et al. 2012; Shukla et al. 2015). However, the current marker-assisted selection techniques are not very promising for GY selections, due to the low proportion of phenotypic GY variance explained by the markers, poor understanding of the genetic control of GY and genotype × environment interactions (*G* × *E*) in response to stress (Snape et al. 2007). Hence, to leverage molecular marker information for complex traits, genomic selection (GS) that incorporates genome-wide marker information to obtain the genomic estimated breeding values of lines was proposed (Haley and Visscher 1998; Meuwissen et al. 2001). In GS, the marker effects are estimated in a “training population” in which the lines are genotyped and phenotyped and subsequently used to predict the breeding value of lines in the ‘validation population’ that have only been genotyped. GS has indeed shifted the paradigm in plant and animal breeding and has the potential to deliver more accurate predictions, reduce cycle time, reduce the cost of phenotyping, and facilitate rapid gains from selection (Muir 2007; Heffner et al. 2009; van der Werf 2009; Jannink et al. 2010; Hayes et al. 2013). While several studies demonstrate the usefulness of GS for complex traits in wheat (Crossa et al. 2014; Battenfield et al. 2016; Hayes et al. 2017; Juliana et al. 2017a, b; Pérez-Rodríguez et al. 2017), one of our key objectives was to evaluate both forward and backward genomic predictions for GY across different nurseries and years, and also explore the potential of utilizing GS in breeding programs.

Reliable, rapid, and inexpensive phenotyping tools will enable breeders to screen large breeding populations for GY in stressed conditions (Reynolds et al. 1999). In this context, high-throughput phenotyping (HTP) of lines that has the potential to improve the accuracy of selections could be useful. Among the physiological HTP traits that can be used for screening adaptation to drought-stressed (DS) environments is canopy temperature, that has been used widely due to its association with GY (Blum et al. 1989; Fischer et al. 1998; Reynolds et al. 1999; Olivares-Villegas et al. 2007; Lopes and Reynolds 2010; Mason and Singh 2014). In addition, spectral reflectances corresponding to different wavelengths of the electromagnetic spectrum that can be remotely measured in a nondestructive manner, integrate the performance of the crop over time periods, help select plants that are physiologically superior, are repeatable and highly heritable can also be used (Aparicio et al. 2000; Araus et al. 2002; Babar et al. 2006a).

The advent of advanced HTP platforms like manned aircraft and unmanned aerial vehicles like drones has enabled automated and precision phenotyping of large breeding populations for their spectral reflectances. These platforms have the potential to revolutionize selections, aid physiological screening, and help gain a better understanding of the genetic control of traits (Cabrera-Bosquet et al. 2012; Prasanna et al. 2013; Andrade-Sanchez et al. 2014; Haghighattalab et al. 2016). The spectral reflectances collected from HTP platforms can be used to build vegetation indices, which help determine the plant’s green biomass, pigment content, water status, etc. (Aparicio et al. 2000; Araus et al. 2002; Babar et al. 2006b). Among the commonly used vegetation indices is the normalized difference vegetation index (NDVI), which is a measure of the plant’s biomass and a reliable indicator of the greenness of plants. It is calculated from the spectral reflectances in the visible (400–700 nm) and near-infrared (700–1300 nm) regions as, NDVI = (near-infrared − visible)/(near-infrared + visible)] (Babar et al. 2006a). It has shown significant association with GY under heat stress and has been used as a screening tool for the heat tolerance of genotypes (Cossani and Reynolds 2012; Lopes and Reynolds 2012).

Indirect selection using correlated responses of high heritability for a target trait of low heritability is an effective breeding strategy in achieving rapid progress for traits of low heritability (Falconer 1960). In this regard, physiological HTP traits can serve as excellent correlated traits for predicting GY and may be particularly useful in early-generation breeding (Reynolds et al. 1999; Montesinos-López et al. 2016, 2018; Rutkoski et al. 2016; Sun et al. 2017). In addition, multivariate genomic prediction for traits of low heritability using correlated traits of high heritability is known to significantly increase the predictive abilities (Jia and Jannink 2012; Fernandes et al. 2017; Okeke et al. 2017). While statistical models that incorporate multiple traits in predictions have been developed, the model-fitting time for large datasets continues to be a significant challenge. A competitive approach that has been proved to be effective in addressing this challenge in several fields is the collaborative-filtering-based recommender system, which has been very successfully used in electronic commerce Web sites like Amazon (Linden et al. 2003), Netflix (Zhou et al. 2008). Recently, the ‘item-based collaborative filtering’ (IBCF) recommender system was applied for multivariate predictions of traits in plant breeding (Montesinos-López et al. 2018). In the IBCF approach, a database of preferences of users for different items is built and for a specific user, *u*_s_, the set of items that the user has rated in the database is considered. This information is then used to compute how similar they are to the target item, followed by the selection of the ‘*k*’ most similar items and computation of similarities between the ‘*k’*-most similar items. A simple weighted average of the similar items is then used to predict the target item for a specific user. Hence, one of our major objectives was to evaluate the IBCF approach for multivariate prediction of GY using a correlated secondary trait (NDVI).

The integration of cutting-edge technologies like GS and HTP has the potential to accelerate gains in breeding for high-yielding climate-resilient wheat varieties. Hence, the goal of this study was to evaluate these tools for potential application in sparse testing GY (minimize one or more years of testing) in the advanced yield-testing stage. For this purpose, we used the elite (second-year) yield trial nurseries (EYT) of CIMMYT’s spring bread wheat for GY evaluated in DS, and late-sown heat-stressed (HS) environments. In addition, our specific objectives were: (1) to assess the phenotypic and genetic correlations of GY in stressed environments with days to heading (DTHD), plant height and green NDVI (GNDVI) (2) to compare genomic and pedigree-based prediction accuracies for GY within and across nurseries/years (3) to compare genomic and pedigree-based predictions in populations with and without full-sibs, and gain a better understanding of the relative advantage of genomic predictions over the pedigree in populations with different family structures (4) to evaluate multivariate predictions of GY using GNDVI with the IBCF approach (5) to evaluate multivariate predictions of GY from GNDVI and relationships (genomic or pedigree) (6) to compare pedigree, genomic, and HTP-based predictions and identify the best approach for practical implementation in various stages of a breeding program.

## Materials and methods

### Populations, field trials, and stress treatment designs

We used four different EYT nurseries comprising 4368 lines that were developed using the selected bulk breeding method and each EYT nursery comprised 1092 *F*_6_:*F*_7_ derived lines. They were planted at the Norman E. Borlaug Research station, Ciudad Obregon, Sonora, Mexico, in the 2013–2014 (EYT 13–14), 2014–2015 (EYT 14–15), 2015–2016 (EYT 15–16) and 2016–2017 (EYT 16–17) growing seasons. Each of these nurseries was sown in 39 trials, and each trial comprised 28 lines and two checks in an alpha lattice design, with three replications and six blocks. For the DS environment, the trials were sown at the optimum planting date (around the last week of November) in raised beds that received 250 mm of water in two irrigations. The late-sown HS environment was simulated by sowing later than the optimal time (around the last week of February) and naturally exposing the lines to high-temperature stress, particularly during the critical grain-filling period. This environment received an optimum of 500 mm of water in five irrigations.

### Phenotypic data

The DTHD (number of days from germination to 50% of spike emergence) and the height of the plant from the ground to the top of the spike in centimeters were recorded for all the lines. Harvested grain weight was calculated on a plot basis and used as a measure of GY. High-throughput phenotyping data were collected with a hyperspectral camera (A-series, Micro-Hyperspec VNIR, Headwall photonics Fitchburg, MA) from the Alava Remote Sensing Spectral Solution (ARS3, Alava Ingenieros, Madrid, Spain), that was mounted in a Piper PA-16 Clipper aircraft (Fig. S1). The camera’s sensor had a 12-bit radiometric resolution, covered the light spectrum in the 400–850 nm region with a 7.5-nm full width at half maximum and the integration time was set to 18 ms. The flights were done around noontime and aligned to the solar azimuth angle at 300 m above the ground, resulting in a ground sampling distance of 30 cm. For each stressed environment, the aerial imageries were taken several days from heading through maturity. The observations were spaced at approximately seven to 10-day intervals, depending on the weather condition and cloudy or windy days were avoided. In the DS environment, HTP data were collected on the EYT 15–16 and EYT 16–17 nurseries and in the HS environment, data was collected on the EYT 14–15 and EYT 15–16 nurseries.

Processing of the hyperspectral images was done using the ARS3 hyproQ software (http://www.grupoalava.com). Radiometric calibration of the sensor was done using coefficients derived from a calibrated uniform light source and an integrating sphere (CSTMUSS2000C Uniform Source System, LabSphere, North Sutton, NH, USA). Dark frame correction was performed for each flight dataset. Atmospheric calibration was performed using irradiance measurements acquired at the beginning and end of each flight using the aerosol optical depth from sun-photometer measurements (Microtops II, Solar Light Company, Glenside, PA) based on the SMARTS (A Simple Model of the Atmospheric Radiative Transfer of Sunshine) simulation model (Gueymard 1995). Finally, ortho-rectification and geo-referencing of the imageries was performed using PARGE (Parametric Geocoding Software, ReSe Applications Schläpfer, Wil, Switzerland, http://www.rese.ch/) based on data from the inertial navigation system attached to the camera (IG-500 N model, SGB systems S.A.S., Carrières-sur-Seine, France), as described in Zarco-Tejada et al. (2013). Hyperspectral reflectance data was extracted from the aerial imageries using the mean value of the pixels inside the central area of each observed plot (0.5 m from the plot borders was excluded). The plots were represented as polygons and adjusted to match the corresponding location of the plots on the imagery for each date. This was followed by the calculation of GNDVI (an indication of the photosynthetic area of the canopy) using the formula: GNDVI = (*R*_780_ − *R*_550_)/(*R*_780_ + *R*_550_) (Gitelson et al. 1996), where ‘*R*’ is the reflectance at the particular wavelength.

### Statistical analysis of the phenotypic data

#### Best linear unbiased estimates (BLUEs) and phenotypic data quality control

The best linear unbiased estimates (BLUEs) for GY and GNDVI for each date of measurement were calculated using the ASREML statistical package (Gilmour 1997), with the following mixed model:

1 $$y_{ijkl} = \mu + g_{i} + t_{j} + r_{k\left( j \right)} + b_{{l\left( {jk} \right)}} + \varepsilon_{ijkl}$$

where *y*_*ijkl*_ is the observed GY or GNDVI, *μ* is the mean, $g_{i}$ is the fixed effect of the genotype, *t*_*j*_ is the random effect of the trial that is independent and identically distributed (IID) $\left( {t_{j} \sim N \left( {0, \sigma_{t}^{2} } \right)} \right)$, $r_{k\left( j \right)}$ is the random effect of the replicate within the trial with IID $\left( {r_{k\left( j \right)} \sim N \left( {0, \sigma_{r}^{2} } \right)} \right)$, $b_{{l\left( {jk} \right)}}$ is the random effect of the incomplete block within the trial and the replicate with IID $\left( {b_{{m\left( {jk} \right)}} \sim N \left( {0, \sigma_{b}^{2} } \right)} \right)$ and *ɛ*_*ijkl*_ is the residual with IID $\left( {\varepsilon_{ijkl} \sim N \left( {0, \sigma_{\varepsilon }^{2} } \right)} \right)$. For across-nursery predictions, GY and GNDVI BLUEs for the different dates were obtained for each genotype by including the random effect of the environment in model (1) (the environments were used as random effects, because we were interested in the non-environment specific performance of the lines).

**Fig. 1:**
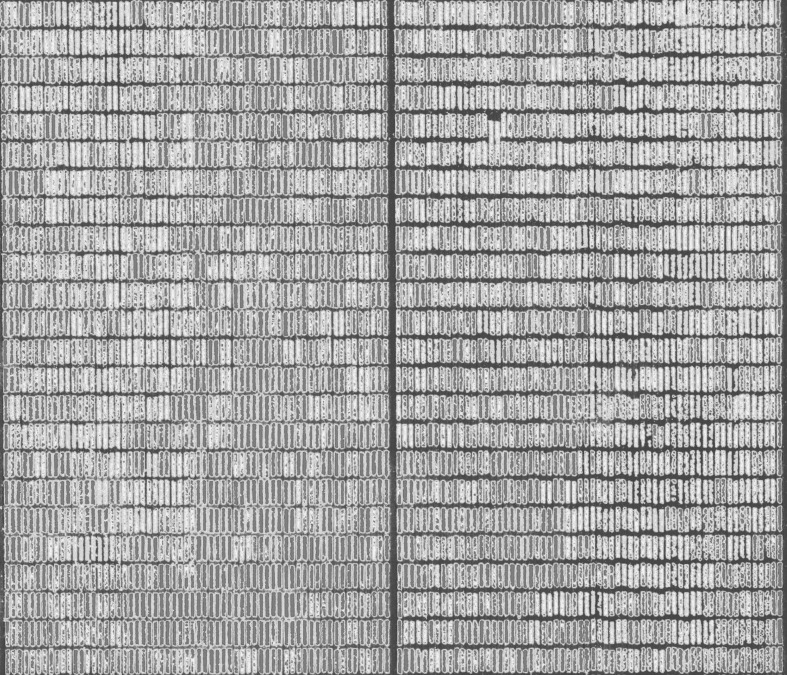
Heatmap of the high-throughput phenotyping derived green normalized difference vegetation index at the grain-filling stage for a subset of lines in an elite yield trial nursery, evaluated in the late-sown heat-stressed environment (red represents high NDVI, green represents low NDVI and blue represents the soil) (colour figure online)

Quality control of the phenotypic data was done by removing the outliers that were detected with the Huber’s robust fit outliers method (Huber and Ronchetti 2009) in the ‘JMP’ statistical software (www.jmp.com). Here, any value more than ‘*K*’ spreads (*K* was set to 4) from the center was considered as missing. For the GNDVI BLUEs, we calculated the across-date heritabilities across dates of measurement and removed the dates with low heritabilities. The GNDVI measurement dates were then classified into heading, grain-filling and maturity growth stages. A heatmap illustrating the range of GNDVI at the grain-filling stage for some lines in an EYT evaluated in the HS environment is shown in Fig. 1.

### Genotyping data

Genome-wide markers for the 4368 lines in the four EYT nurseries were obtained using genotyping-by-sequencing (GBS) (Elshire et al. 2011; Poland et al. 2012) done at Kansas State University, using an Illumina HiSeq 2500 with 190 samples pooled per lane. The TASSEL 5 (Trait Analysis by aSSociation Evolution and Linkage) GBS v2 pipeline (Glaubitz et al. 2014) was used to call marker polymorphisms, and the minor allele frequency for single nucleotide polymorphism (SNP) discovery was set to 0.01. The markers were anchored to the International Wheat Genome Sequencing Consortium’s (IWGSC) first version of the reference sequence (RefSeq v1.0) assembly of the bread wheat variety *Chinese Spring* (The International Wheat Genome Sequencing Consortium 2018). The alignment of 6,075,743 unique tags with Bowtie2 (Langmead and Salzberg 2012) resulted in the overall alignment of 63.98% of the tags, with 28.92% unique alignments and 35.06% multiple alignments. The SNPs at each locus were then tested for three criteria (1) Inbred coefficient: > 80% (2) Fisher Exact test: *p* < 0.001 (3) Chi-square: < 9.21 [the Chi-square value was used to check whether the number of observed genotype classes fitted the expected number of genotype classes (96%) due to inbreeding and the critical Chi-square value at two degrees of freedom and alpha of 0.01 was 9.21]. The 78,662 SNPs that passed at least one of these filters were further filtered for greater than 50% missing data, less than 5% minor allele frequency and greater than 5% percent heterozygosity, and we obtained 9285 markers. Imputation of missing marker data was done using LinkImpute (Money et al. 2015) in TASSEL (Bradbury et al. 2007) version 5. The lines that had greater than 50% missing data were also removed, and 3485 lines were obtained (766 lines in EYT 13–14, 775 lines in EYT 14–15, 964 lines in EYT 15–16 and 980 lines in EYT 16–17). The genotyping and phenotyping datasets for all the lines are provided in Supplementary File 1.

#### Estimation of phenotypic correlations, heritability, and genetic correlations

The phenotypic Pearson correlation coefficients between DTHD, plant height, GY and GNDVI were obtained. We also calculated the square root of the broad-sense heritability (*H*) for GY and GNDVI across replicates, within each nursery on a line-mean basis using the formula,

2 $$H = \sqrt {\frac{{\sigma_{g}^{2} }}{{\sigma_{g}^{2} + \frac{{\sigma_{\varepsilon }^{2} }}{\text{nreps}}}}}$$

where *σ*_*g*_^2^ is the genetic variance, *σ*_*ɛ*_^2^ is the error variance, and nreps is the number of replications. The estimates of genetic and residual variances were obtained using the average information-restricted maximum likelihood algorithm (Gilmour et al. 1995) implemented in the ‘*R*’ package ‘heritability’(Kruijer et al. 2015).

The genetic correlations between DTHD, plant height, GY, and GNDVI were obtained using the ‘*R*’ package EMMREML (Akdemir and Okeke 2015) with the ‘emmremlMultivariate’ function. This function solves a multivariate Gaussian mixed model that has a known covariance structure and can be represented as:

3 $$\varvec{Y}^{\text{T}} = \varvec{X\beta }^{\text{T}} + \varvec{Za}^{\text{T}} + {\varvec{\upvarepsilon}}^{\text{T}}$$

where $\varvec{Y}^{\text{T}} = \left[ {\varvec{Y}_{1} , \ldots ,\varvec{Y}_{t} } \right],\varvec{\beta}^{\text{T}} = \left[ {\varvec{\beta}_{1} , \ldots ,\varvec{\beta}_{t} } \right], \varvec{a}^{\text{T}} = \left[ {\varvec{a}_{1} , \ldots ,\varvec{a}_{t} } \right], {\varvec{\upvarepsilon}}^{\text{T}} = \left[ {{\varvec{\upvarepsilon}}_{1} , \ldots ,{\varvec{\upvarepsilon}}_{t} } \right],$ where *t* is the number of traits, $\varvec{Y}_{1}$ to $\varvec{Y}_{t}$ are vectors of BLUEs (GY and GNDVI at different dates of measurement) or phenotypic values (DTHD, height) of *n* genotypes, **X** is the design matrix of fixed effects, **Z** is the design matrix of random effects, $\varvec{\beta}_{1}$ to $\varvec{\beta}_{t}$ are vectors of fixed effects for traits one to *t*, **a**_1_ to **a**_t_ are vectors of random effects for traits one to *t* and ${\varvec{\upvarepsilon}}_{1}$ to ${\varvec{\upvarepsilon}}_{\text{t}}$ are vectors of residuals for traits one to *t*. Here, the distribution of $\varvec{a}$ is multivariate normal with *N*(0_(*n*×*t*)×1_, **V**_G_ ⊗ **K**), where **V**_G_ is the *t* × *t* additive genetic (co)variance matrix of traits, ⊗ denotes the Kronecker product and **K** is the known relationship matrix of order *n* × *n*. The distribution of ${\varvec{\upvarepsilon}}$ is multivariate normal with *N*(0_(*n*×*t*)×1_, **V***ɛ* ⊗ **I**_n_), where **V***ɛ* is the residual (co)variance matrix of traits and **I**_*n*_ is the *n* × *n* identity matrix.

### Design of training and validation populations

#### Training and validation populations for within-nurseries predictions

For within-nursery predictions, we used all the lines in the nurseries, subsets of lines with and without full-sibs and also lines within a narrow range of DTHD in the following designs (Table 1):

**Table 1:** Training and validation populations for within elite yield trial nursery (EYT) predictions

	All the lines in a nursery				A subset of lines in each nursery within a narrow range of days to heading (drought-stressed environment)				A subset of lines in each nursery within a narrow range of days to heading (late-sown heat-stressed environment)				Lines that are represented by only one progeny per cross and have no full-sibs				Lines with at least one other full-sib in the population			
Elite yield trial nursery	13–14	14–15	15–16	16–17	13–14	14–15	15–16	16–17	13–14	14–15	15–16	16–17	13–14	14–15	15–16	16–17	13–14	14–15	15–16	16–17
Population size	766	775	964	980	488	561	730	665	535	668	763	743	342	226	243	201	260	399	539	613
Training population	613	620	771	784	390	449	584	532	428	534	610	594	274	181	194	161	208	319	431	490
Validation population	153	155	193	196	98	112	146	133	107	134	153	149	69	45	49	40	52	80	108	123

1. *All the lines in a nursery* In this design, lines from both within and across-families were considered. The lines in each EYT nursery were divided into fivefold, and four of them were used as the training population to predict the remaining lines in the fivefold or the validation population.
2. *Lines that are represented by only one progeny per cross and have no full*-*sibs* In this design, we used a subset of each of the nurseries comprising only one progeny per cross and performed cross-validations.
3. *Lines with at least two other full*-*sibs in a nursery* We evaluated the advantage of having full-sibs in the training population by using a subset of lines that had at least two other full-sibs in that nursery. We then used 50% of the full-sibs in the training population to predict the other 50% of full-sibs in the validation population.
4. *Lines within a narrow range of days to heading in each nursery* Since DTHD was moderately correlated with GY in several datasets, we created subsets of lines in each nursery by excluding the lines that were at the tails of the DTHD distributions and including only those that were within the standard deviation for DTHD. In the DS environment, the range of DTHD in the subsets was 78–87 days (EYT 13–14), 74–81 days (EYT 14–15), 79–85 days (EYT 15–16) and 70–79 days (EYT 16–17). Similarly, in the HS environment, the range of DTHD in the subsets was 59–66 days (EYT 13–14), 52–59 days (EYT 14–15), 55–61 days (EYT 15–16) and 56–62 days (EYT 16–17).

#### Design of training and validation populations for across-nurseries predictions

In predictions across nurseries, we performed both forward and backward predictions using one nursery as the training population to predict the other, for every possible nursery combination. In addition, we also used all the other three EYTs to predict any given EYT. The across-nursery predictions were done using all the lines in the nurseries and also using only a subset of lines in each nursery within a narrow range of DTHD.

#### Univariate predictions of grain yield using genomic and pedigree-based relationships

While several models are available for genomic predictions, their similarities have been reported in previous studies (Heslot et al. 2012; Rutkoski et al. 2012; Juliana et al. 2017b). So, we used only the genomic best linear unbiased prediction (GBLUP) model with the genomic relationship matrix (***G***-matrix) calculated from markers (VanRaden 2008). The GBLUP model was implemented in the ‘*R*’ package BGLR (Pérez and de los Campos 2014) and can be represented as,

4 $$y_{i} = \mu + u_{i} + \varepsilon_{i}$$

where *y*_*i*_ the response variable for individual *i*, *μ* is the general mean, $u_{i} \varvec{ }$ is the additive genetic effect for individual *i* assuming that the joint distribution of the vector of additive genetic effects **u** is *N* (**0**, ***G****σ*_g_^2^), where ***G*** is the additive relationship matrix and *σ*_g_^2^ is the variance component associated with markers and *ε*_i_ is the error term assuming that the joint distribution of **ε** is *N* (0, **I***σ*_e_^2^), where *σ*_e_^2^ is the residual variance.

For pedigree-based predictions, the genomic relationship matrix in model (4) was replaced with the pedigree relationship matrix (***A***-matrix) which captures the identity-by-descent relationships. In addition, we also fitted model (4) with the combined genomic and pedigree-based relationships (***G-*** and ***A***-matrices). The Pearson’s correlation between the GY BLUEs and the predicted breeding values was used as a measure of prediction accuracy for all the models. The prediction accuracies were not divided by the square root of the heritability, because the genotypes were not replicated across the years and the across-year heritabilities could not be estimated.

### Multivariate predictions of grain yield

#### Multivariate predictions of grain yield using the green normalized difference vegetation index

We used the IBCF approach for multivariate prediction of GY using its similarity to GNDVI measured at different dates. In the IBCF approach, the following expression is used to predict the rating $P_{{i,j^{{\prime }} }}$ for user *i* in item $j^{{\prime }}$ (Sarwar et al. 2001),

5 $$P_{{i,j^{\prime}}} = \frac{{\mathop \sum \nolimits_{j\epsilon N} y_{i,j} w_{{j,j^{\prime}}}}}{{\mathop \sum \nolimits_{j\epsilon N} \left| {w_{{j,j^{\prime}}}} \right|}}$$

Here, the summation is over all other rated items ($j\epsilon N$) for user *i* where *N* is the total number of rated items, $w_{{j,j^{{\prime }} }}$ is the weight between items *j* and $j^{{\prime }}$, and *y*_*i*,*j*_ is the rating for user *i* on item *j*. An item-to-item similarity matrix incorporating similarity between the items was built using the cosine similarity $\cos \left( \theta \right) = \frac{{\mathop \sum \nolimits_{{\varvec{j} = 1}}^{\varvec{n}} \varvec{x}_{\varvec{j}} \varvec{y}_{\varvec{j}} }}{{\sqrt {\mathop \sum \nolimits_{{\varvec{j} = 1}}^{\varvec{n}} \varvec{x}_{\varvec{j}}^{2} } \sqrt {\mathop \sum \nolimits_{{\varvec{j} = 1}}^{\varvec{n}} \varvec{y}_{\varvec{j}}^{2} } }}$), and used to obtain the weights used in Eq. (5) (Montesinos-López et al. 2018). We implemented IBCF for both within and across-nursery predictions in the complete set of lines and also in the subset of lines that had a narrow range of DTHD using the ‘*R*’ package IBCF.MTME (Luna-Vazquez et al. 2018).

#### Multivariate prediction of grain yield using the green normalized difference vegetation index and relationships (genomic or pedigree)

We performed multivariate genomic (***G***-matrix) and pedigree-based (***A***-matrix) predictions using the model described in (3), along with the correlated responses (GNDVI BLUEs measured at different dates) using the ‘*R*’ package EMMREML (Akdemir and Okeke 2015).

## Results

### Phenotypic data analysis

The phenotypic means and ranges of traits in the two stressed (DS and HS) environments were analyzed (Table S1). In the DS environment, average GY was the highest in EYT 16–17 (4.8 t/ha) and ranged between 2.2 and 5.8 t/ha across the four nurseries. Similarly, DTHD ranged from 59 to 96 days, and plant height ranged from 63 to 106 cm across the different nurseries. In the HS environment, average GY was the highest in EYT 14–15 (3.8 t/ha) and ranged between 1 and 5.5 t/ha in the four nurseries. The DTHD ranged between 45 and 69 days, and plant height ranged between 48 and 88 cm in the HS environment. We also analyzed the phenotypic GY variance in the full-sib families within each nursery (Fig. S2) and observed that the deviation of the full-sibs from the family mean was low and ranged between 0.06 and 1.34 t/ha in both the environments.

### Phenotypic correlations, genetic correlations and heritabilities of traits

**Fig. 2:**
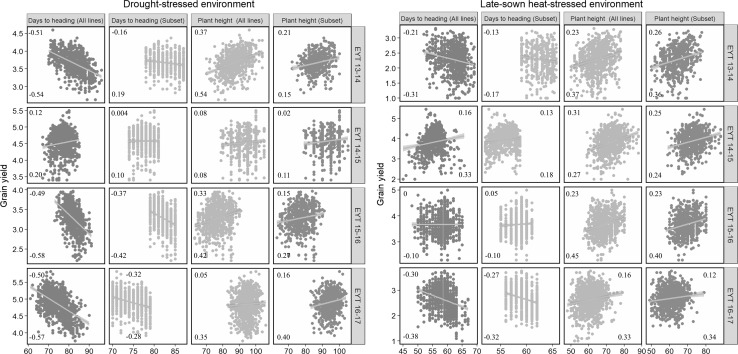
Correlations of grain yield with days to heading, and plant height in the drought-stressed and late-sown heat-stressed environments in the elite yield trial (EYT) nurseries. The values on the upper corner represent the phenotypic Pearson’s correlations, and the values on the lower corner represent the genetic Pearson’s correlations

The phenotypic and genetic correlations of GY with DTHD and plant height were analyzed for all the lines in the nurseries and also for the subset of lines within a narrow range of DTHD (Fig. 2). In the DS environment, phenotypic and genetic correlations of DTHD with GY were moderate and negative (ranged between − 0.49 and − 0.58) in three nurseries, but slightly positive in EYT 14–15. Plant height had a positive low to moderate correlation with GY (ranged between 0.05 and 0.54).

In the HS environment, phenotypic and genetic correlations of DTHD with GY were weak to moderately negative in EYT 13–14 and EYT 16–17, close to zero in EYT 15–16 and slightly positive in EYT 14–15. Plant height had positive correlations with GY in all the four nurseries that ranged from 0.16 to 0.45. The *p* values for the test of significance of the correlations indicated that most correlations, except GY with DTHD and plant height in EYT 14–15 of the DS environment and GY with DTHD in EYT 15–16 of the HS environment were significant at the threshold level of 0.001. Overall, the phenotypic and genetic correlations of GY with DTHD and plant height in the subset of lines were lower than the correlations in the complete set of lines in both the environments.

We also analyzed the phenotypic and genetic correlations of GY, DTHD, and plant height across the DS and HS environments (Table S2). For GY in the DS and HS environments, the phenotypic correlations ranged between 0.12 and 0.31, while the genetic correlations ranged between 0.16 and 0.36. But, DTHD had high genetic correlations (ranged between 0.87 and 0.91), and plant height had moderately high genetic correlations across these environments (ranged between 0.51 and 0.59).

The phenotypic and genetic correlations of GNDVI measured at different dates with GY in the complete set and subset of lines were analyzed (Table 2). In the DS environment, the highest phenotypic and genetic correlations of GY with GNDVI in EYT 15–16 (− 0.35 and − 0.51) and EYT 16–17 (− 0.44 and − 0.50) were observed around mid-March (that coincided with the late grain-filling stage or the maturity stage depending on whether the genotype was early or late) in the complete set of lines. However, in the subset of lines within a narrow range of DTHD, the correlations between GNDVI and GY were lower with a maximum genetic correlation of − 0.29 in EYT 15–16 and − 0.24 in EYT 16–17. In the HS environment, the highest phenotypic and genetic correlations of GY with GNDVI in EYT 14–15 (0.54 and 0.70) and EYT 15–16 (0.58 and 0.61) were observed during the grain-filling stage in the complete set of lines. Similarly, in the subset of lines, the highest phenotypic and genetic correlations between GY and GNDVI in EYT 14–15 (0.60 and 0.70) and EYT 15–16 (0.63 and 0.63) were also observed during the grain-filling stage and were slightly higher.

**Table 2:** Phenotypic and genetic Pearson correlation coefficients of the green normalized difference vegetation index (GNDVI) with grain yield in the drought and late-sown heat-stressed environments of the two elite yield trial (EYT) nurseries

Nursery	Date of phenotyping	Growth stage	Number of lines^a^	Correlation with GY	
				Phenotypic	Genetic
*Drought-stressed environment*					
EYT 15–16	February 26th, 2016	Grain-filling 1	964	− 0.09	− 0.24
			730	0.08	− 0.04
	March 3rd, 2016	Grain-filling 2	964	− 0.16	− 0.36
			730	0.02	− 0.11
	March 15th, 2016	Maturity/grain-filling 3	964	− 0.35	− 0.51
			730	− 0.18	− 0.29
EYT 16–17	January 23rd, 2017	Vegetative	980	− 0.23	− 0.23
			665	− 0.11	− 0.03
	February 10th, 2017	Heading	980	− 0.41	− 0.44
			665	− 0.20	− 0.09
	February 16th, 2017	Grain-filling 1	980	− 0.42	− 0.45
			665	− 0.22	− 0.12
	March 15th, 2017	Maturity/grain-filling 2	980	− 0.44	− 0.50
			665	− 0.20	− 0.24
*Late-sown heat-stressed environment*					
EYT 14–15	April 14th, 2015	Vegetative	775	0.52	0.59
			668	0.54	0.59
	April 28th, 2015	Grain-filling 1	775	0.55	0.66
			668	0.60	0.66
	May 6th, 2015	Grain-filling 2	775	0.54	0.70
			668	0.60	0.70
EYT 15–16	May 2nd, 2016	Grain-filling 1	964	0.58	0.61
			763	0.63	0.63
	May 9th, 2016	Grain-filling 2	964	0.54	0.54
			763	0.60	0.56

*EYT* elite yield trial, *GY* grain yield, *DTHD* days to heading, *GNDVI* green normalized difference vegetation index

^a^The number of lines refers to all the lines in a nursery or a subset of lines in each nursery within a narrow range of days to heading

The line-mean broad-sense heritabilities for GY ranged between 0.73 and 0.80 in the DS environment and between 0.65 and 0.91 in the HS environment (Table S3). Similarly, the line-mean broad-sense heritabilities for GNDVI in the different dates of measurement ranged between 0.77 and 0.97 in the DS environment and between 0.75 and 0.94 in the HS environment.

### Genomic and pedigree relationships

The ***A***-matrices and ***G***-matrices for all the 3485 lines in the different nurseries were rescaled between zero and one and visualized by heat maps (Fig. S3). We observed a higher degree of relationships with the ***G***-matrix compared to the ***A***-matrix, which was most likely due to the realized relationships and the identity-by-state similarities in genomic regions under selection pressure captured by the ***G***-matrix. In addition, there was no grouping of nurseries based on the genomic relationships indicating the existence of some genetic relatedness across the nurseries.

### Grain yield prediction accuracies

1. *Genomic and pedigree*-*based prediction accuracies for grain yield within and across nurseries*
  The GY prediction accuracies for the complete set of lines in the nurseries and the subset of lines within a narrow range of DTHD were analyzed (Fig. 3). In the DS environment, the average cross-validation accuracies for GY within nurseries were 0.50 ± 0.06 with genomic predictions, 0.49 ± 0.07 with pedigree-based predictions and 0.55 ± 0.06 with the combined genomic and pedigree-based predictions in the complete set of lines. Similarly, in the HS environment, the average within-nursery accuracies were 0.51 ± 0.04 with genomic predictions, 0.46 ± 0.03 with pedigree-based predictions and 0.53 ± 0.03 with the combined genomic and pedigree-based predictions. The within-nursery prediction accuracies in the subset were similar to the prediction accuracies in the complete set of lines across all the models. Overall, genomic predictions resulted in accuracies that were similar to or slightly higher than the pedigree-based prediction accuracies, with a maximum increase of 0.10. The combined markers and pedigree-based model resulted in the best within-nursery prediction accuracies that were on average 0.04 higher than the genomic prediction accuracies.

**Fig. 3:**
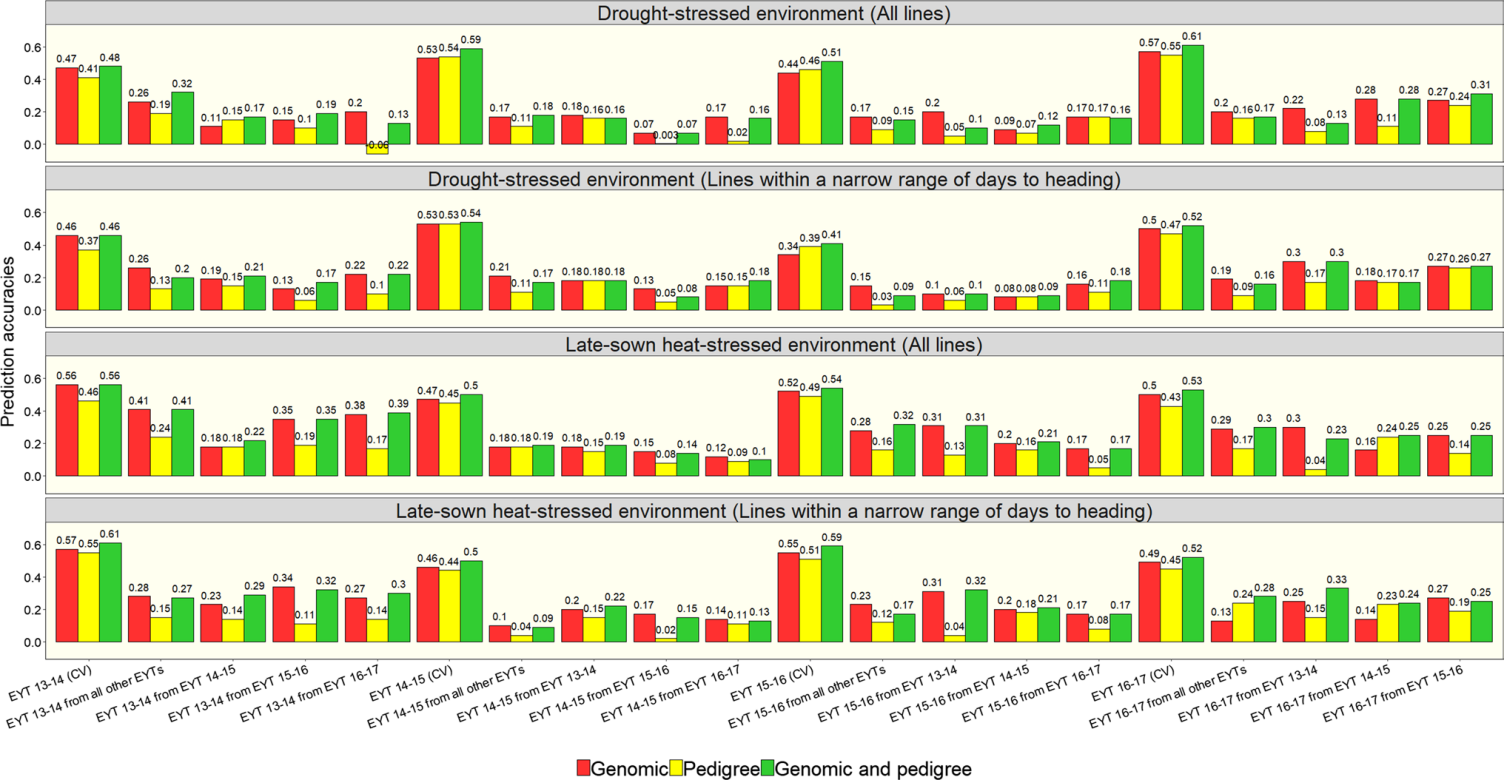
Genomic, pedigree and combined genomic and pedigree-based prediction accuracies for grain yield in the drought-stressed and late-sown heat-stressed environments of the elite yield trial (EYT) nurseries using all the lines in the nurseries and only a subset of lines within a narrow range of days to heading. The within-nursery cross-validations (CV) are represented by the nursery (i.e., EYT 13–14, EYT 14–15, EYT 15–16 and EYT 16–17), and the across-nursery predictions are represented by the nursery and the nursery that was used to predict it (i.e., EYT 13–14 from EYT 14–15)
  In across-nursery predictions, the average accuracies in the DS environment for the complete set and the subset of lines were 0.18 ± 0.06 with genomic predictions, 0.11 ± 0.07 with pedigree-based predictions and 0.17 ± 0.07 with the combined genomic and pedigree-based predictions. Similarly, in the HS environment, the average across-nursery accuracies were 0.23 ± 0.08 with genomic predictions, 0.14 ± 0.06 with pedigree-based predictions and 0.24 ± 0.08 with the combined genomic and pedigree-based predictions. Genomic predictions outperformed pedigree-based predictions across nurseries in most datasets and the average increase in accuracies by using genomic predictions over pedigree-based predictions was 0.07 ± 0.06 (maximum increase of 0.26) in the DS environment and 0.11 ± 0.07 (maximum increase of 0.27) in the HS environment.
  We also observed that the combined markers and pedigree-based model did not have a significant advantage over genomic predictions across nurseries. While the accuracies were similar in many datasets, the average increase in accuracies from the combined model over genomic predictions was only 0.02 ± 0.02 and 0.03 ± 0.04 in the DS and HS environments, respectively. We also observed a decrease in accuracies using the combined marker and pedigree-based model over the GBLUP, whenever the pedigree-based model had lower accuracies than the GBLUP. When all other EYTs were used to predict a given EYT nursery, the accuracies were generally similar or slightly lower than the predictions from single nurseries in both the environments, except in a few datasets. While the average increase in accuracies with three nurseries in the training population versus a single nursery was 0.04 ± 0.03 (maximum increase of 0.13), the average decrease in accuracies was 0.08 ± 0.04 (maximum decrease of 0.17).
2. *Genomic and pedigree*-*based prediction accuracies for grain yield in populations with different family structures*
  Genomic and pedigree-based prediction accuracies in populations with and without full-sibs in the complete set of lines were analyzed (Table 3). In populations with only one progeny per cross, the average genomic prediction accuracies were 0.31 ± 0.12, and the average pedigree-based prediction accuracies were 0.24 ± 0.11 in the DS environment. Similarly, in the HS environment, the average genomic prediction accuracies were 0.32 ± 0.12, and the average pedigree-based prediction accuracies were 0.22 ± 0.01. Overall, genomic predictions performed similar to the pedigree-based predictions in 50% of the datasets with only one progeny per cross and higher than the pedigree-based predictions in other datasets, resulting in increase in accuracies ranging between 0.09 and 0.20 in both the environments.

**Table 3:** Genomic prediction accuracies for grain yield in drought-stressed and late-sown heat-stressed environments with and without full-sibs in the training population

Nursery	Relationship matrix	Cross-validation in populations with only one progeny per cross		Prediction of 50% of full-sibs from the other 50% of full-sibs	
		Drought-stressed	Late-sown heat-stressed	Drought-stressed	Late-sown heat-stressed
EYT 13–14	Genomic	0.42 ± 0.07	0.43 ± 0.08	0.52 ± 0.02	0.64 ± 0.04
	Pedigree	0.33 ± 0.14	0.24 ± 0.05	0.51 ± 0.02	0.62 ± 0.04
EYT 14–15	Genomic	0.28 ± 0.12	0.21 ± 0.11	0.55 ± 0.02	0.56 ± 0.02
	Pedigree	0.16 ± 0.08	0.21 ± 0.14	0.63 ± 0.02	0.58 ± 0.03
EYT 15–16	Genomic	0.16 ± 0.07	0.42 ± 0.16	0.57 ± 0.02	0.63 ± 0.04
	Pedigree	0.14 ± 0.11	0.22 ± 0.15	0.59 ± 0.0	0.60 ± 0.03
EYT 16–17	Genomic	0.38 ± 0.16	0.22 ± 0.11	0.61 ± 0.02	0.58 ± 0.01
	Pedigree	0.34 ± 0.09	0.21 ± 0.14	0.61 ± 0.02	0.50 ± 0.0

*EYT* elite yield trial
  When 50% of the full-sibs were included in the training population, the average genomic prediction accuracies were 0.56 ± 0.04, and the average pedigree-based prediction accuracies were 0.59 ± 0.06 in the DS environment. In the HS environment, the average genomic prediction accuracies were 0.60 ± 0.04, and the average pedigree-based prediction accuracies were 0.58 ± 0.05. Overall, the genomic prediction accuracies were similar to the pedigree-based prediction accuracies across all the datasets in this design.
  We also observed that the genomic prediction accuracies in populations with full-sibs in the training population were on average 0.27 ± 0.10 higher than the accuracies in populations with no full-sibs/only one progeny per cross, resulting in 24–256% increase in accuracies in both the environments. Similarly, pedigree-based prediction accuracies in populations with full-sibs in the training population were on average 0.35 ± 0.10 higher than the accuracies in populations with only one progeny per cross, resulting in 54–321% increase in accuracies in both the environments.
3. *Multivariate prediction of grain yield using the green normalized difference vegetation index*
  We used GNDVI in an IBCF approach to predict GY and observed that the average cross-validation accuracies in the DS environment were 0.41 in the complete set of lines and 0.22 in the subset of lines within a narrow range of DTHD (Table 4). But, in the HS environment, the average cross-validation accuracies were 0.58 in the complete set and 0.63 in the subset of lines. In across-nursery predictions, the average accuracies were: 0.39 and 0.20 for the complete set and subset of lines in the DS environment and 0.56 and 0.62 for the complete set and subset of lines in the HS environment, respectively.

**Table 4:** Multivariate prediction of grain yield from the green normalized difference vegetation index, genomic and pedigree-based relationships

Environment	Prediction approach	All the lines in a nursery				A subset of lines in each nursery within a narrow range of days to heading			
		EYT 15–16 (within-nursery CV)	EYT 16–17 (within-nursery CV)	Prediction of EYT 15–16 from EYT 16–17	Prediction of EYT 16–17 from EYT 15–16	EYT 15–16 (within-nursery CV)	EYT 16–17 (within-nursery CV)	Prediction of EYT 15–16 from EYT 16–17	Prediction of EYT 16–17 from EYT 15–16
Drought-stressed	GNDVI in an IBCF approach	0.36 ± 0.02	0.45 ± 0.02	0.36	0.41	0.20 ± 0.02	0.24 ± 0.02	0.19	0.20
	Genomic and GNDVI	0.51 ± 0.05	0.58 ± 0.03	0.11	0.34	0.40 ± 0.07	0.50 ± 0.07	0.18	0.21
	Pedigree and GNDVI	0.51 ± 0.03	0.55 ± 0.04	0.08	0.17	0.41 ± 0.05	0.47 ± 0.06	0.04	0.07

		EYT 14–15 (within-nursery CV)	EYT 15–16 (within-nursery CV)	Prediction of EYT 14–15 from EYT 15–16	Prediction of EYT 15–16 from EYT 14–15	EYT 14–15 (within-nursery CV)	EYT 15–16 (within-nursery CV)	Prediction of EYT 14–15 from EYT 15–16	Prediction of EYT 15–16 from EYT 14–15
Late-sown heat-stressed	GNDVI in an IBCF approach	0.58 ± 0.01	0.57 ± 0.03	0.56	0.56	0.62 ± 0.01	0.63 ± 0.01	0.61	0.63
	Genomic and GNDVI	0.59 ± 0.05	0.59 ± 0.05	0.39	0.15	0.57 ± 0.05	0.61 ± 0.06	0.34	0.18
	Pedigree and GNDVI	0.63 ± 0.03	0.54 ± 0.05	0.18	0.04	0.60 ± 0.03	0.58 ± 0.04	0.28	0.05

*EYT* elite yield trial, *GY* grain yield, *GNDVI* green normalized difference vegetation index
4. *Multivariate prediction of grain yield using the green normalized difference vegetation index and relationships* (*genomic or pedigree*)
  We evaluated the ability of GNDVI combined with relationships (genomic or pedigree) to predict GY (Table 4). In the DS environment, the average cross-validation accuracies within nurseries using genomic relationships and GNDVI were 0.55 and 0.45 in the complete set and subset of lines, respectively. Similarly, with the pedigree relationships and GNDVI, the cross-validation accuracies in the complete set and the subset of lines were and 0.53 and 0.44, respectively. In across-nursery GY predictions, the average accuracies were 0.23 and 0.20 using genomic relationships with GNDVI and 0.13 and 0.06 using pedigree relationships with GNDVI in the complete set and the subset of lines, respectively.

In the HS environment, the average cross-validation accuracy within nurseries was 0.59 in the complete set and subset of lines, using both genomic and pedigree-based relationships combined with GNDVI. In across-nursery predictions for the HS environment, the average accuracies were 0.27 and 0.26 using genomic relationships with GNDVI and 0.11 and 0.17 using pedigree relationships with GNDVI in the complete set and the subset of lines, respectively.

## Discussion

### Phenotypic data, phenotypic and genetic correlations among traits

We analyzed GY in four different EYT nurseries and observed that the DS environment had higher average GY (4.1 t/ha), compared to the HS environment (3.1 t/ha). Since the average GY under optimum conditions was 6.4 t/ha, it was reduced by 36% in the DS environment and 52% in the HS environment, thereby emphasizing the importance of breeding for resilience to these stresses. In the DS environment, plants headed almost at the same time as in the optimum environment but were on average 22 days earlier in the HS environment. This is due to the high temperatures and longer day length in this environment that are known to result in shorter cycles, quicker maturity, and accelerated crop development rates. These are considered as adaptations of plants to hot weather enabling them to avoid excessive stress (Zahedi and Jenner 2003; Fischer 2011; Semenov et al. 2014; Trnka et al. 2014; Mondal et al. 2016). The plants in the stressed environments were also shorter than in the optimal environment (average height reduction of 15 cm, and 35.5 cm in the DS and HS environments, respectively).

We also observed that GY in both the stressed environments was negatively correlated with DTHD and positively correlated with plant height, as observed previously (Mondal et al. 2016). However, nursery EYT 14–15 evaluated in the DS environment was an exception to this general trend, where GY had no correlations with DTHD and plant height, because of the warmer temperatures observed in this year. A linear decrease in GY with increasing DTHD in the DS environment was also observed and indicated that in populations with a wide variation for DTHD, irrigation cut-off time is a key determinant of the sensitivity of genotypes to the stress (Fischer and Maurer 1978).

Analysis of associations between GNDVI measured in different growth stages and GY indicated that the highest phenotypic and genetic correlations with GY were observed during the grain-filling stage in the DS environment and were negative. While GNDVI is generally positively correlated with GY, because of it being an indicator of the greenness or biomass of the plants, it was negatively correlated with GY in the DS environment as also observed previously (Rutkoski et al. 2016). This is most likely due to the range of DTHD in the populations used (the late lines were greener and had a lower yield). The decrease in correlations between GNDVI and GY in the subset of lines within a narrow range of days to heading, indicates that the higher correlations between these traits in the complete set of lines are most likely due to the broader range of DTHD and its correlation with GY. In the HS environment, the highest correlations of GNDVI with GY were positive and coincided with grain-filling, indicating it to be a critical stage in determining GY.

### Grain yield predictions in the stressed environments

This study evaluated GY predictions in empirical data from CIMMYT’s bread wheat breeding program, and our results indicate that genomic predictions perform similar to or slightly better than the pedigree-based predictions within nurseries, and the combined marker and pedigree-based predictions resulted in the highest accuracies as also observed in several other studies (de los Campos et al. 2009; Pérez et al. 2010; Burgueño et al. 2012; Bartholomé et al. 2016; Juliana et al. 2017a, b). In across-nursery predictions, genomic predictions outperformed pedigree-based predictions, and there was no advantage of combining markers and pedigree. We also observed no improvement in prediction accuracies by increasing the number of lines/nurseries in the training population for across-nursery predictions. While this has also been observed in previous studies (Dawson et al. 2013; Lorenz and Smith 2015), substantial *G* × *E* interaction and difference in marker effects for GY in different nurseries/years might explain why a higher number of lines in the training population did not boost the prediction accuracies. Hence, predictions from large training populations/multiple nurseries might be useful only in cases when the environments or years are correlated with the predicted year.

In populations with no full-sibs in the training population, we observed that the genomic prediction accuracies were either similar or resulted in 27.3–91% increase in accuracies over the pedigree-based prediction accuracies. But, in populations with at least one full-sib in the training population, the genomic and pedigree-based prediction accuracies were similar. This indicates that the high accuracies obtained using the pedigree-based predictions are primarily due to, (1) the full-sibs in the training population (2) the minimal phenotypic GY variance among the full-sibs resulting from phenotypic GY selection in the first-year yield trials and (3) the minimal Mendelian sampling variance among the full-sibs, because of very small family sizes. These results indicate that GS will not be advantageous over pedigree-based selections in the EYT stage, given the family structure in these nurseries, highlighting the importance of implementing GS at the appropriate stage in the breeding program, where there is sufficient Mendelian sampling variation between the sibs. Nevertheless, predictions in populations with full-sibs in the training population compared to populations with only one progeny per cross, resulted in 24–321% increase in accuracies, clearly implying that genetic relatedness between the training and validation populations is crucial for good predictions as also reported in previous studies (Habier et al. 2010; Clark et al. 2012; Pszczola et al. 2012; Thorwarth et al. 2017).

The successful application of the IBCF recommender system in multivariate prediction of GY using GNDVI was demonstrated in this study. For within-nursery predictions in the DS environment, the accuracies using GNDVI in an IBCF approach were only slightly lower than the univariate predictions using genomic/pedigree-based relationships. However, in the subset of lines within a narrow range of DTHD, we observed a decrease in accuracies using GNDVI indicating that the predictability of GY in the complete set of lines was mostly due to the confounding effect of DTHD. In the HS environment, using GNDVI in an IBCF approach resulted in accuracies that were slightly higher than the genomic/pedigree-based prediction accuracies in the complete set of lines, and a slight improvement in accuracies was also observed in the subset. While the IBCF approach is very competitive in terms of its speed compared to many other multivariate models, it can be effectively applied in scenarios where trait correlations with GY are moderate to high.

Overall, for within-nursery predictions in the DS environment, the strategies that resulted in the highest accuracies were the genomic and pedigree-based prediction models that had 50% of full-sibs in the training population. In addition, the combined models including, (1) genomic relationships and GNDVI (2) pedigree relationships and GNDVI (3) genomic and pedigree relationships also resulted in high accuracies. However, in EYT 16–17, the GBLUP and pedigree models also performed well and resulted in accuracies that were only slightly lower than the combined models. In the HS environment, the models with genomic/pedigree relationships and GNDVI, GNDVI in an IBCF approach and the genomic and pedigree models that had 50% of full-sibs in the training population resulted in the highest within-nursery accuracies. While Rutkoski et al. (2016) and Sun et al. (2017) reported that secondary traits increased within-nursery GY accuracies by 70% in prediction models, the maximum increase that we obtained by integrating GNDVI was only 25% in genomic prediction models and 40% in pedigree-based prediction models. The scenarios in which GS and HTP can be successfully implemented in breeding for GY within-nurseries/years are, (1) when the training population has a reasonable number of full-sibs (2) when the HTP trait is highly correlated with GY. However, considering the cost, an integrated approach with the pedigree and HTP will be the most cost-effective for implementation at this advanced yield-testing stage used in this study.

In across-nursery predictions, the best accuracies in the DS and HS environments were obtained using GNDVI in an IBCF approach. It is interesting that the relationship based predictions across years were not more advantageous than using GNDVI of the validation population in the prediction models, although the lines in the different nurseries had some genetic relatedness. This also highlights the importance of having some information on a line’s performance in a particular environment (a correlated trait like GNDVI in this case) for successfully predicting GY and sparse testing should be considered cautiously when breeding for widely adapted and stable lines for diverse target environments.

### Challenges for implementing genomic and high-throughput phenotyping based selections in breeding for grain yield

This study has identified several challenges for implementing GS and HTP in breeding for GY that are discussed below:

1. *Genotype × environment interactions*
  The moderate to poor GY prediction accuracies across nurseries/years in this study result from the low heritability of the trait, vagaries of the weather, *G* × *E* interactions, non-homogeneity of stress conditions across years that can increase error variance, variations in management and soil heterogeneity (Blum 1988, 2005; Lopes et al. 2014). However, even in controlled irrigations, non-uniform test conditions are not uncommon, because of the difficulty in uniform application of small quantities of water (Calhoun et al. 1994). In addition, climatic variability including temperature and precipitation variations account for about a third of the yield variability (Ray et al. 2015), and changes in these variables are known to affect crop yields and shift the phenology (Tao et al. 2006; Mondal et al. 2016; Albers et al. 2017) as also observed in this study. Therefore, the complexity of plant responses to stressed conditions resulting from *G* × *E* interactions, the difficulties in determining all possible genetic responses to all possible combinations of environments and the inability to phenotype large breeding populations avoiding confounding weather effects (Reynolds and Tuberosa 2008) hamper accurate predictions of GY. One potential approach to address this challenge would be to integrate crop growth models with genomic selection models (Rincent et al. 2017).
2. *Confounding effects of phenology and stress escape*
  Both, DTHD and plant height often confound screening tolerance to drought and heat stress, and unless populations controlled for these traits are used, only major genes associated with these traits will be associated with stress adaptations (Reynolds and Tuberosa 2008; Reynolds et al. 2009; Fleury et al. 2010; Lopes et al. 2014). In our study, the chromosomal locations of the loci significantly associated with GY in nurseries where DTHD had a high correlation coincided with the vernalization and photoperiod sensitivity genes (unpublished results). While these genes underlie adaptation of wheat to different environments (Yan et al. 2004; Cane et al. 2013), they also confound the expression of stress–adaptive minor genes and reduce our ability to distinguish between high GY due to adaptation to the stress and high GY by avoiding stress. The ability of genotypes that flower and mature early to escape drought by rapid phenological development and shorter life-cycle is referred to as ‘drought escape’ (Fischer and Maurer 1978; Blum 1988, 2005). It has also been advocated as a strategy to breed for drought-tolerant plants by reducing the coincidence of the sensitive stages of plant development with the stress (Loss and Siddique 1994; Blum 1996; Araus et al. 2002; Olivares-Villegas et al. 2007; Pinto et al. 2010). Similarly, genotypes that head early and have longer post-heading duration are considered to be tolerant to heat stress (Talukder et al. 2014; Mondal et al. 2016).

Breeders avoid the confounding effects of DTHD and stress escape by comparing GY of early and late lines with appropriate early and late check varieties. However, for both genomic and HTP-based predictions, it is important to control the effects of DTHD. So, we used subsets of lines within a narrow range of DTHD in this study and observed similar accuracies as in the complete set. However, several other approaches have been suggested to address this challenge that include: (1) using a covariate or a correction factor to adjust for DTHD (Fischer and Maurer 1978) (2) evaluating GS on early and late sub-populations and removing extremes of DTHD (Reynolds et al. 2009) (3) evaluating GS on lines that have a restricted range of DTHD of approximately 1 week (Reynolds et al. 1994) (4) using populations that are not segregating for major genes (Pinto et al. 2010) or characterizing populations for the major genes (vernalization, photoperiod sensitivity and plant height) and only using individuals monomorphic for these genes (Lopes et al. 2014) (5) using populations with a restricted range of DTHD for estimating marker effects like the Seri/Babax recombinant inbred line population (Olivares-Villegas et al. 2007), and the wheat association mapping initiative population (Lopes et al. 2015). While these options should be considered in designing training populations for prediction of GY in stressed environments, it should be emphasized that the effects of DTHD will be inevitable when data from large breeding populations is used for training the models.

### Opportunities for implementing genomic and high-throughput phenotyping based selections in breeding for grain yield

The promising cross-validation accuracies obtained in this study indicate several opportunities for implementing GS and HTP within nurseries/years, some of which are discussed below:

1. *Minimizing replications*
  Breeding programs that have multiple and expensive within-site replications can use GS- or HTP-based selections to minimize them if there is at least one reliable replication for training prediction models, as also observed in Juliana et al. (2018). The cost–benefits of minimizing replications will depend on the cost of phenotyping per unit of yield trial replication versus the cost of (1) tissue sampling and deoxyribonucleic acid extraction (2) genotyping (3) hyperspectral camera and the aerial platform (4) HTP data collection (5) data management and analysis. In addition, the time involved in the logistics of genotyping, collecting HTP data and processing HTP images should be minimal to facilitate implementation and quick selection decisions.
2. *Scaling*-*up selections to larger nurseries within years that are not yield*-*tested in stressed environments*
  While this study has evaluated cross-validations within the second-year yield trials, these EYT nurseries can also be used as training sets to predict the first-year yield trial nurseries (about 9000 lines) grown in the same year. These nurseries are generally not phenotyped for GY under stressed environments in CIMMYT, because of the cost, labor and resource constraints. Hence, GS- and/or HTP-based predictions of GY in two stressed environments might result in cost-savings of about 180, 000 USD (assuming that the cost of a yield trial plot is 10 USD). However, given that the cost of genotyping 9000 samples (180,000 USD, at the cost of 20 USD per sample) is almost similar, real cost-gains can be achieved only when (1) breeding programs have higher GY phenotyping costs and (2) the cost of genotyping is lower or shared for predicting other traits. Nevertheless, phenotyping large populations also requires vast land area that might not be available for many breeding programs and GS can be a powerful selection tool in such a case.
3. *Scaling*-*up selections to earlier generations that are not yield*-*tested*
  While this study has applied the IBCF approach on nurseries of about 1000 lines, our ultimate objective was to scale this approach for predicting GY with HTP traits in several ten-thousands of *F*_5_:*F*_6_ derived lines that are grown in small plots and not yield-tested or are too expensive for genotyping. This could result in huge cost-savings, if the HTP measurements for lines in the small plots are well correlated with their HTP measurements and yields in large plots.
4. *Increasing accuracies in early*-*generation across*-*family selections*
  The HTP trait based predictions can also be used to increase the selection accuracy in early-generation across-family selections. While within-family selections are not possible in early generations, HTP traits can enable selection of the best families for GY in stressed environments, that can then be advanced.

## Conclusion

In conclusion, predicting GY in stressed environments across years is challenging due to its complexity, plasticity, and *G* × *E* interactions. Hence, some information on a genotype’s or a related individual’s phenotypic performance in a particular environment is important for training prediction models and minimizing risks. In addition, assessment of the cost–benefits by integrating GS and HTP in a breeding program is critical for the effective implementation of these technologies. Nevertheless, this study has successfully evaluated GS- and HTP-based predictions in CIMMYT’s EYT nurseries, and we conclude that they can be integrated in increasing the size of populations screened and evaluating unphenotyped large nurseries within years.

## Electronic supplementary material

Below is the link to the electronic supplementary material.

Fig. S1 The high-throughput phenotyping platform comprising a hyperspectral camera (A-series, Micro-Hyperspec VNIR, Headwall photonics Fitchburg, MA) from the Alava Remote Sensing Spectral Solution (ARS3, Alava Ingenieros, Madrid, Spain) (right), mounted in a Piper PA-16 Clipper aircraft (left) (TIFF 6068 kb)

Fig. S2 Phenotypic variance for grain yield within the full-sib families of each elite yield trial (EYT) nursery evaluated in drought-stressed and late-sown heat-stressed environments (TIFF 13125 kb)

Fig. S3 Heatmaps of the pedigree and genomic relationship matrices for all the lines in the four elite yield trial (EYT) nurseries (TIFF 34450 kb)

Supplementary material 4 (CSV 71011 kb)

Supplementary material 5 (DOCX 16 kb)
