# Supplementary figures and images for: Integrating genomic-enabled prediction and high-throughput phenotyping in breeding for climate-resilient bread wheat
Source: Theor Appl Genet. 2018 Oct 19;132(1):177–94. doi: 10.1007/s00122-018-3206-3 (PMC6320358; doi:10.1007/s00122-018-3206-3)

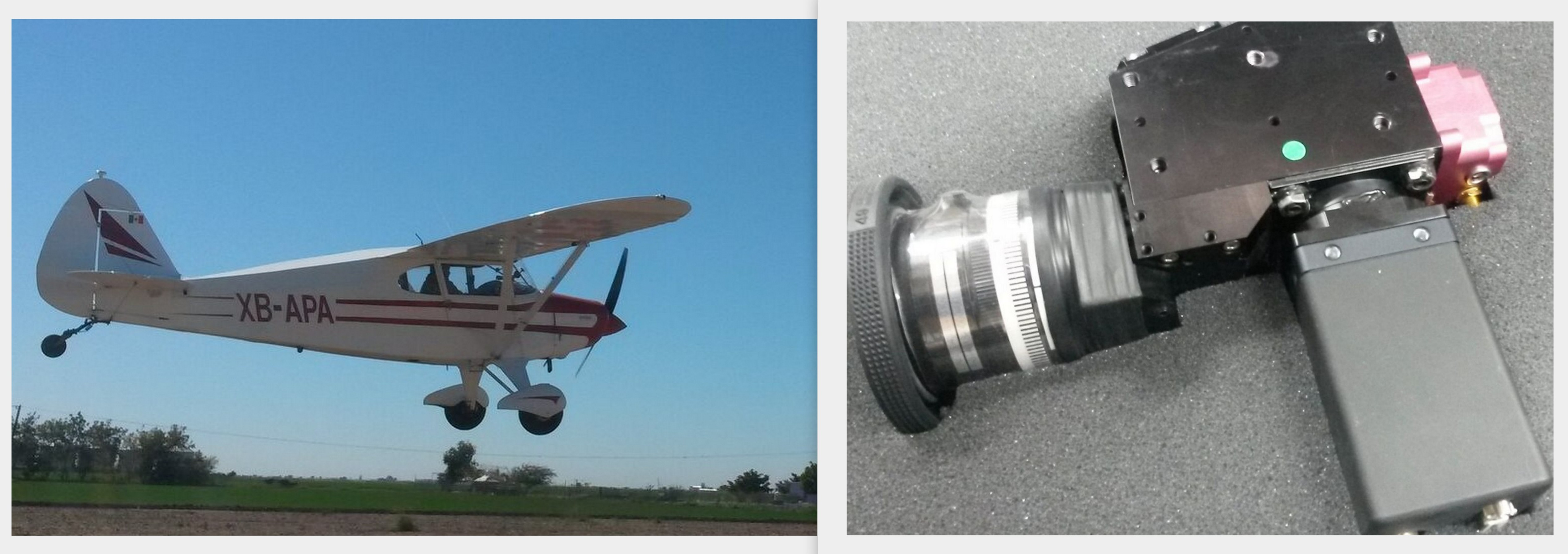

Supplement: Supplementary file 1 — The high-throughput phenotyping platform comprising a hyperspectral camera (A-series, Micro-Hyperspec VNIR, Headwall photonics Fitchburg, MA) from the Alava Remote Sensing Spectral Solution (ARS3, Alava Ingenieros, Madrid, Spain) (right), mounted in a Piper PA-16 Clipper aircraft (left) (TIFF 6068 kb) [file 122_2018_3206_MOESM1_ESM.tiff]

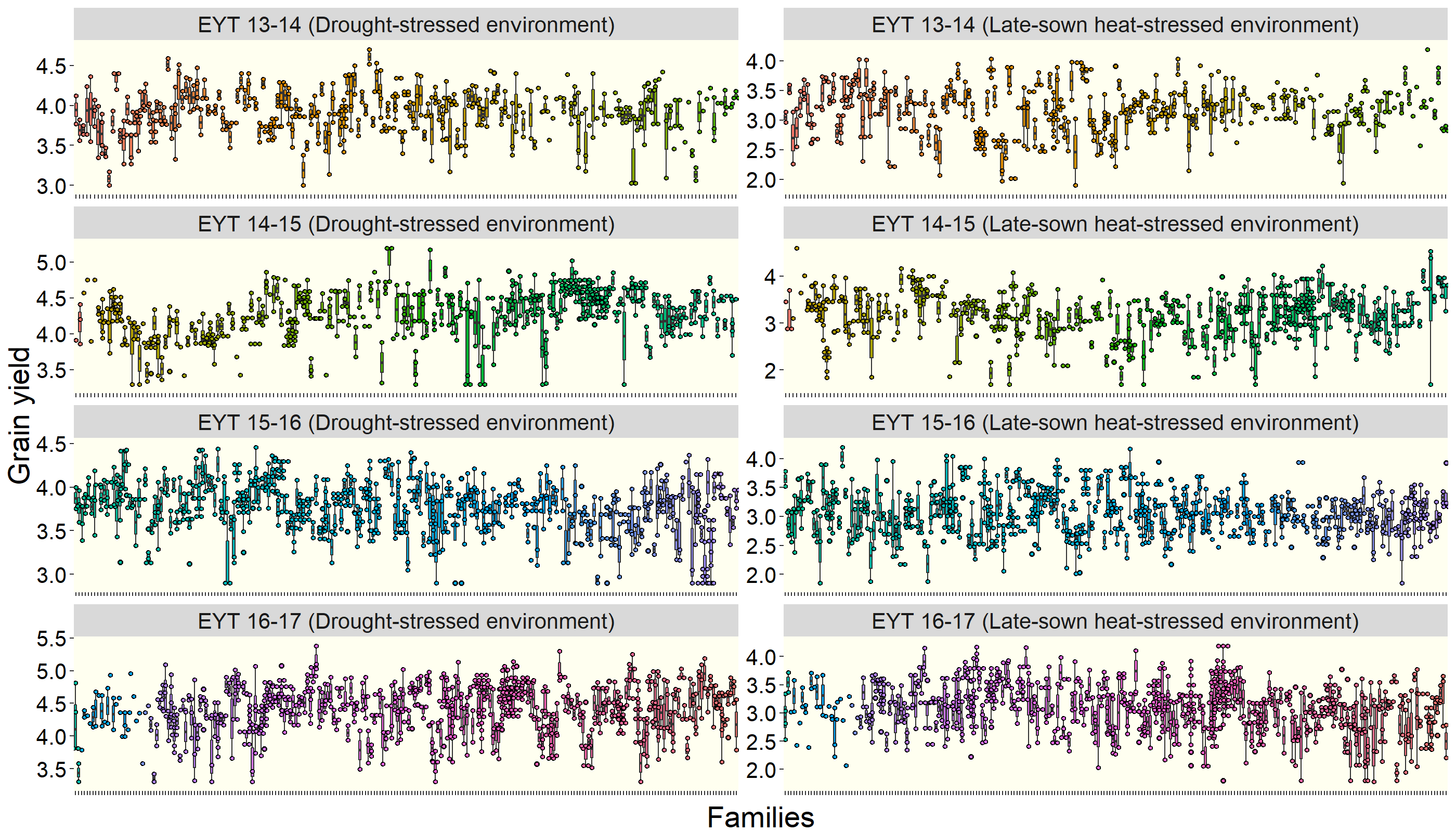

Supplement: Supplementary file 2 — Phenotypic variance for grain yield within the full-sib families of each elite yield trial (EYT) nursery evaluated in drought-stressed and late-sown heat-stressed environments (TIFF 13125 kb) [file 122_2018_3206_MOESM2_ESM.tiff]

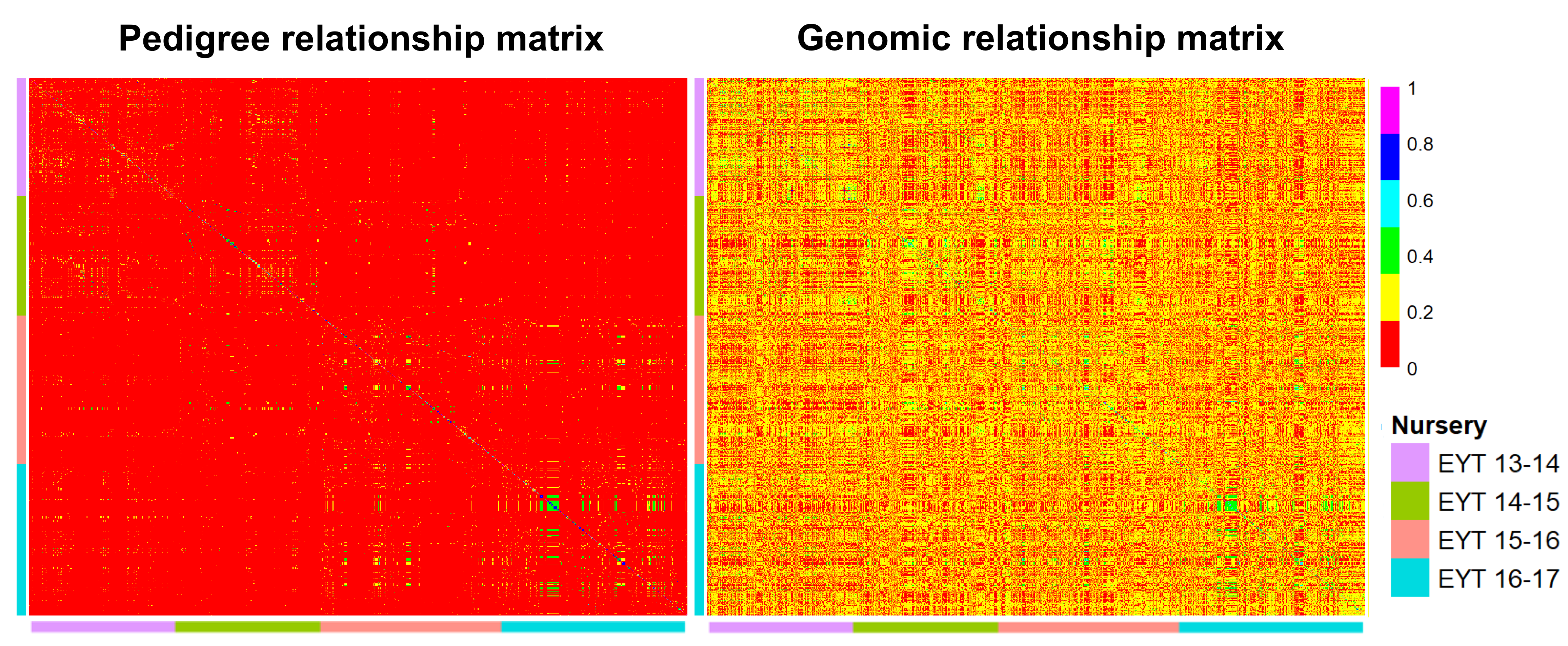

Supplement: Supplementary file 3 — Heatmaps of the pedigree and genomic relationship matrices for all the lines in the four elite yield trial (EYT) nurseries (TIFF 34450 kb) [file 122_2018_3206_MOESM3_ESM.tiff]
