# Supplementary material for: Integrating genomic-enabled prediction and high-throughput phenotyping in breeding for climate-resilient bread wheat
Source: Theor Appl Genet. 2018 Oct 19;132(1):177–94. doi: 10.1007/s00122-018-3206-3 (PMC6320358; doi:10.1007/s00122-018-3206-3)
Supplement: Supplementary file 5 — Supplementary material 5 (DOCX 16 kb) [file 122_2018_3206_MOESM5_ESM.docx]

**Table S1: Mean and range of grain yield, days to heading, and plant height in the drought and late-sown heat-stressed environments of the elite yield trial (EYT) nurseries**

| Environ-  ment | Traits | EYT 13-14 | | EYT 14-15 | | EYT 15-16 | | EYT 16-17 | |
| --- | --- | --- | --- | --- | --- | --- | --- | --- | --- |
|  |  | Mean | Range | Mean | Range | Mean | Range | Mean | Range |
| Drought stressed | Grain yield (t/ha) | 3.7 + 0.3 | 2.6 - 4.6 | 4.5 + 0.4 | 3.4 - 5.5 | 3.3 + 0.3 | 2.2 - 4 | 4.8 + 0.4 | 3.6 - 5.8 |
|  | Days to heading | 82 + 5.5 | 70 - 96 | 77.6 + 3.6 | 67 - 85 | 82 + 3 | 73 - 91 | 74.6 + 5 | 59 - 93 |
|  | Plant height (cm) | 87.1 + 6 | 70 - 104 | 93 + 5.3 | 79 - 105 | 77 + 5.2 | 63 - 91 | 94.5 + 4 | 83 - 106 |
| Late-sown heat-stressed | Grain yield (t/ha) | 2.3 + 0.4 | 1 - 3.3 | 3.8 + 0.5 | 2 - 5.5 | 3.7 + 0.4 | 2.3 - 5 | 2.6 + 0.5 | 1 - 3.8 |
|  | Days to heading | 61.9 + 3.5 | 52 - 69 | 55.4 + 2.7 | 45 - 65.7 | 58 + 3 | 48 - 65 | 58.8 + 3 | 50 - 67 |
|  | Plant height (cm) | 64.8 + 5.7 | 50 - 78 | 72.7 + 5 | 59 - 88 | 68.9 + 4.5 | 57 - 80 | 60.9 + 4.8 | 48 - 79 |

EYT – Elite yield trial

**Table S2: Phenotypic and genetic Pearson correlation coefficients of grain yield, days to heading, and plant height in the drought-stressed and late-sown heat-stressed environments of the elite yield trial (EYT) nurseries**

| Nursery | Trait | GY in HS | | DTHD in HS | | Height in HS | |
| --- | --- | --- | --- | --- | --- | --- | --- |
|  |  | Phenotypic | Genetic | Phenotypic | Genetic | Phenotypic | Genetic |
| EYT 13-14 | GY in DS | 0.25 | 0.36 | -0.44 | -0.47 | 0.05 | 0.31 |
|  | DTHD in DS | -0.19 | -0.26 | 0.86 | 0.87 | 0.08 | 0 |
|  | Plant height in DS | 0.16 | 0.39 | -0.49 | -0.60 | 0.31 | 0.54 |
| EYT 14-15 | GY in DS | 0.21 | 0.25 | 0.08 | 0.19 | 0.01 | 0 |
|  | DTHD in DS | 0.23 | 0.39 | 0.85 | 0.91 | 0.04 | 0.15 |
|  | Plant height in DS | -0.03 | -0.15 | -0.11 | -0.06 | 0.33 | 0.59 |
| EYT 15-16 | GY in DS | 0.12 | 0.16 | -0.38 | -0.53 | -0.05 | 0.12 |
|  | DTHD in DS | 0.05 | -0.07 | 0.77 | 0.89 | 0.03 | -0.22 |
|  | Plant height in DS | -0.0007 | 0.18 | -0.27 | -0.51 | 0.25 | 0.53 |
| EYT 16-17 | GY in DS | 0.31 | 0.34 | -0.36 | -0.51 | 0.10 | 0.36 |
|  | DTHD in DS | -0.24 | -0.21 | 0.75 | 0.87 | -0.12 | -0.31 |
|  | Plant height in DS | -0.02 | 0.08 | 0.04 | -0.26 | 0.26 | 0.51 |

EYT – Elite yield trial, GY – grain yield, DTHD – days to heading, DS – drought-stressed, HS – late-sown heat-stressed

**Table S3: Additive genetic variance, error variance and line-mean broad-sense heritabilities for grain yield and green-normalized difference vegetation index in the drought-stressed and late-sown heat-stressed environments of the elite yield trial (EYT) nurseries**

| Trait | Variance components and heritability estimates | Drought-stressed | | | | Late-sown heat-stressed | | | |
| --- | --- | --- | --- | --- | --- | --- | --- | --- | --- |
|  |  | EYT 13-14 | EYT 14-15 | EYT 15-16 | EYT 16-17 | EYT 13-14 | EYT 14-15 | EYT 15-16 | EYT 16-17 |
| Grain yield | $\sigma_{g}^{2}$ | 0.24 | 0.20 | 0.28 | 0.27 | 0.59 | 0.78 | 0.25 | 0.55 |
|  | $\sigma_{\varepsilon}^{2}$ | 0.06 | 0.07 | 0.09 | 0.07 | 0.06 | 0.14 | 0.13 | 0.08 |
|  | H | 0.80 | 0.73 | 0.76 | 0.79 | 0.91 | 0.85 | 0.65 | 0.87 |
| GNDVI, Date 1 | $\sigma_{g}^{2}$ |  |  | 0.002 | 2.46e-04 |  | 0.0005 | 0.002 |  |
|  | $\sigma_{\varepsilon}^{2}$ |  |  | 6.27e-05 | 0.0002 |  | 0.00019 | 0.0001 |  |
|  | H |  |  | 0.97 | 0.89 |  | 0.75 | 0.94 |  |
| GNDVI, Date 2 | $\sigma_{g}^{2}$ |  |  | 0.0015 | 4.39e-04 |  | 0.0054 | 8.96e-04 |  |
|  | $\sigma_{\varepsilon}^{2}$ |  |  | 4.99e-05 | 0.0003 |  | 0.0004 | 0.0007 |  |
|  | H |  |  | 0.97 | 0.91 |  | 0.93 | 0.88 |  |
| GNDVI, Date 3 | $\sigma_{g}^{2}$ |  |  | 9.39e-04 | 5.18e-04 |  | 0.0009 |  |  |
|  | $\sigma_{\varepsilon}^{2}$ |  |  | 0.0007 | 0.0003 |  | 7.65e-05 |  |  |
|  | H |  |  | 0.89 | 0.91 |  | 0.93 |  |  |
| GNDVI, Date 4 | $\sigma_{g}^{2}$ |  |  |  | 0.001 |  |  |  |  |
|  | $\sigma_{\varepsilon}^{2}$ |  |  |  | 0.0003 |  |  |  |  |
|  | H |  |  |  | 0.77 |  |  |  |  |

$\sigma_{g}^{2}$- Additive genetic variance; $\sigma_{\varepsilon}^{2}$- Error variance; H - broad-sense heritability, GNDVI – green normalized difference vegetation index
